# ANATOMIC DAMAGE OF THE LOWER ESOPHAGEAL SPHINCTER AFTER SUBTOTAL GASTRECTOMY

**DOI:** 10.1590/0102-672020210002e1633

**Published:** 2022-01-31

**Authors:** Owen KORN, Attila CSENDES, Patricio BURDILES, Enrique LANZARINI, Ana HENRÍQUEZ

**Affiliations:** 1 Department of Surgery, Clinical Hospital University of Chile, Santiago, Chile

**Keywords:** Gastroesophageal reflux, Esophageal sphincter, lower, Gastrectomy, Refluxo gastroesofágico, Esfíncter esofágico inferior, Gastrectomia

## Abstract

Dysfunction of the lower esophageal sphincter (LES), gastroesophageal reflux disease, and erosive esophagitis in patients undergoing subtotal gastrectomy are commonly recognized occurrences, but until now the causes remain unclear.

**AIM:**

The hypothesis of this study is that subtotal gastrectomy provokes changes on the LES resting pressure and its competence, due to the anatomical damage of it, given that the oblique “Sling” fibers, one of the muscular components of the LES, are transected during this surgical procedure.

**METHODS:**

Seven adult mongrel dogs (18-30 kg) were anesthetized and admitted for transection of the proximal stomach. Later, the proximal gastric remnant was closed by a suture. Intraoperatively, slow pull-through LES manometries were performed on each dog, under basal conditions (with the intact stomach), and in the closed proximal gastric remnant. The mean of these measurements is presented, with each dog serving as its control.

**RESULTS:**

The mean LES pressure (LESP) measured in the proximal gastric remnant, compared with the LESP in the intact stomach, was decreased in five dogs, increased in one dog, and remained unchanged in other dogs.

**CONCLUSION:**

The upper transverse transection of the stomach and closing the stomach remnant by suture provoke changes in the LESP. We suggested that these changes in the LESP are secondary to transecting the oblique “Sling” fibers of the LES, one of its muscular components. The suture and closing of the proximal gastric remnant reanchor these fibers with more, less, or the same tension, whether or not modifying the LESP.

## RESUMO - RACIONAL

Disfunção do esfíncter esofágico inferior (EEI), doença do refluxo gastroesofágico e esofagite erosiva em pacientes submetidos à gastrectomia subtotal são ocorrências comumente reconhecidas, mas até agora as causas permanecem obscuras.

**OBJETIVO:**

A hipótese deste estudo é que a gastrectomia subtotal provoque alterações na pressão de repouso do EEI e na sua competência, devido ao dano anatômico desta, visto que as fibras oblíquas “Sling”, um dos componentes musculares do EEI, são seccionadas durante este procedimento cirúrgico.

**MÉTODOS:**

Sete cães adultos sem raça definida (18-30 kg) foram anestesiados e submetidos à transecção do estômago proximal. Em seguida, o remanescente gástrico proximal foi fechado por sutura. No intraoperatório, manometria lenta foi realizada em cada cão, em condições basais (com estômago intacto) e no remanescente gástrico proximal fechado. A média dessas medidas é apresentada, com cada cão servindo como seu próprio controle.

**RESULTADOS:**

A pressão média do EEI medida no remanescente gástrico proximal, em comparação com a pressão do EEI no estômago intacto, foi diminuída em cinco cães, aumentada em um cão e sem alterações no outro cão.

**CONCLUSÃO:**

A secção transversa superior do estômago e o fechamento do remanescente do estômago por sutura provocam alterações na pressão do EEI. Sugerimos que essas mudanças na pressão do EEI são secundárias à secção das fibras oblíquas “Sling” do esfíncter, um de seus componentes musculares. A sutura e o fechamento do remanescente gástrico proximal, reancora essas fibras com mais, menos ou a mesma tensão, modificando ou não a pressão do EEI.

## INTRODUCTION

In older and recent surgical literature, it is well recognized that a percentage of patients undergoing distal or subtotal gastrectomy may develop esophagitis or gastroesophageal reflux disease (GERD) postoperatively^1^ ^,^ ^4^ ^,^ ^10^ ^,^ ^11^ ^,^ ^15^ ^,^ ^19^ ^,^ ^25^. Some authors have suggested that this is due to a dysfunction of the lower esophageal sphincter (LES), and although there are several theories, until now the cause of this dysfunction is not clear^10^ ^,^ ^11^ ^,^ ^14^ ^,^ ^24^ ^,^ ^26^.

In this experimental study, our hypothesis is that subtotal gastrectomy can modify LES pressure (LESP) as a consequence of anatomical damage to the LES, since the transverse transection of the stomach at the upper middle third cuts the oblique muscular fibers of the LES (Sling fibers), which are one of the muscular components of the LES^17^.

## METHODS

This experimental study was performed in seven adult mongrel dogs (2 females and 5 males) weighing 20-30 kg. This animal model has been used historically in experimental manometric studies of the LES, because the regional anatomy of the LES in dogs is quite comparable to human anatomy and, therefore, constitutes a validated model^6^ ^,^ ^8^ ^,^ ^18^ ^,^ ^21^. Institutional Animal Care Protocols for Research were strictly followed, and particular care was taken to prevent any pain or stress on the animals. After an 8-h fast, the dogs were anesthetized with intravenous thiopental sodium, intubated, and finally oxygenated by controlled ventilation. No muscle relaxant was given, and a bolus of 5-μg/kg fentanyl was administered for pain control.

**Figure 1 -:**
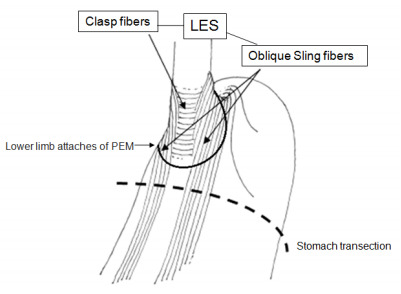
Schematic representation of lower esophageal sphincter (LES), the short semicircular “clasp” fibers on the lesser curve at the cardia, and the long oblique “Sling” fibers, extending from the cardia at the great curvature side, run in parallel to the lesser curve into the gastric antrum. The dotted line shows the level of stomach transection in experimental model, distal to the lower attachment of phrenoesophageal membrane (PEM).

Special care was taken to maintain satisfactory oxygenation, normal blood pressure, and minimal bleeding to avoid any effect of these factors over LESP^3^ ^,^ ^16^. After a midline laparotomy, stationary manometry of the LES was performed in all dogs under two conditions; the first one, with the stomach intact, was considered the baseline measurement. Thereafter, the stomach was transected transversely at the upper third, starting at the lesser curve, 3 cm distal to the gastric insertion of the phrenoesophageal membrane (PEM; Figure 1). After the gastric remnant was closed with a running one-layer suture, the second manometry was performed.

These manometric studies were performed using a device with four polyvinyl catheters joined together with the side distal holes arranged radially at the same level and with four proximal openings spaced every 5 cm (Synectics Medical, Stockholm, Sweden). They were perfused continuously with 0.9% NaCl from a hydropneumocapillary pump at a rate of 0.5 ml/min (Arndorfer Medical Specialties, Milwaukee, WI, USA) and connected to a computer manometry system with four channels (Synectics Medical). Before each test, the system was calibrated, and the occlusion of any side hole produced an increase of 400 mmHg within 1 s. In each condition, three slow pull-through withdrawals were performed. Due to the asymmetry of sphincter pressures, each withdrawal provided different pressures in each channel and, therefore, the average of these four values was considered the final LESP for each withdrawal. Fundic gastric pressure was considered a zero reference. In the analysis of the results, each dog had its control.

## RESULTS

The first manometric recording was the LES resting pressure intraoperatively for each dog and was taken as baseline, that is, with the abdomen open but with the stomach intact. The observed LESP ranged between 9.6 and 15.9 mmHg in six animals. One dog showed a basal pressure which was double these values (30.5 mmHg, Table 1).

**Table 1 -:** Lower esophageal sphincter pressure measurement in each dog under experimental conditions (each dog had its control).

Dog	Intact stomach LESP (mmHg), mean±SD	Closed gastric remnant LESP (mmHg), mean±SD
1	15.5±2.1	11.7±4.0
2	10.2±0.7	7.5±1.0
3	13.3±0.4	5.0±2.0
4	15.9±1.8	11.6±2.8
5	14.5±5.0	4.3±0.6
6	9.6±1.4	9.3±3.0
7	30.5±1.0	32.5±3.5

**Figure 2 -:**
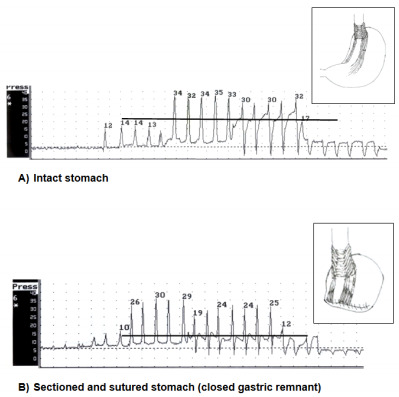
Lower esophageal sphincter pressure (LESP) tracings obtained in experimental conditions intraoperatively (dog 4): (A) LESP in the basal condition with intact stomach; (B) a decrease in pressure after the transection of the stomach and closure of the gastric remnant.

After closing the proximal gastric remnant with a continuous suture, a new manometry was performed, and changes in LESP were registered in each dog and were compared with basal pressure of the same dog. The final mean LESP observed was decreased in five dogs (Figure 2), remained unchanged in one dog (dog 6), and was greater than its baseline in one dog (dog 7).

## DISCUSSION

This experimental study was designed to evaluate eventually changes in the LESP in dogs after transecting the proximal stomach, trying to reproduce the method in other reports in the literature that have studied LESP in patients before and after undergoing a distal gastrectomy^10^ ^,^ ^11^ ^,^ ^14^ ^,^ ^19^ ^,^ ^22^ ^,^ ^24^ ^,^ ^26^.

Historically, a much-argued question that persists today has been to understand how the LES functions and to understand and accept its unique anatomy^7^. For a long time, the existence of an anatomical LES has been questioned, because the regional anatomy does not appear to show a muscle structure that satisfies the arbitrary definition of a sphincter, as a muscle “ring”^2^.

Thus, without an anatomical correlation, but with the clear manometric demonstration of a gastroesophageal sphincter, a discussion started by Fyke et al.^5^ in 1956 concerning the existence of a “physiological sphincter,” which made our understanding of its behavior more difficult. Liebermann-Meffert in the late 1970s confirmed what ancient anatomists had shown, that is, in the cardia, a muscle structure like a ring does not exist, but there are two muscle bands arranged perpendicularly at the cardia: the LES muscle^17^. Despite this detailed anatomical description, the LES has remained conceptually in the mind of many researchers as a muscular ring. This misinterpretation of LES anatomy has prevented the understanding and explanation of the peculiarities of the behavior of this sphincter with its particular anatomical structure^20^.

The cause of development of GERD and/or esophagitis in some patients undergoing distal partial gastrectomy remains unclear^1^ ^,^ ^4^ ^,^ ^10^ ^,^ ^11^ ^,^ ^15^ ^,^ ^19^ ^,^ ^23^ ^,^ ^25^. Some authors have suggested that resection of the distal stomach causes changes in the levels of certain gastrointestinal hormones that would modify the LESP^24^. Others argue that the injury of the PEM, as well as changes in the esophagogastric angle (His) related to the reconstruction of gastrointestinal continuity, may alter the sphincter function^4^ ^,^ ^14^ ^,^ ^19^ ^,^ ^24^ ^,^ ^25^, with some attributing the origin of this LES dysfunction to the type of reconstruction, whether it is a type Billroth I or II^4^ ^,^ ^11^.

However, the explanations offered above are not convincing. It has been established that gastroesophageal reflux or sphincter dysfunction does not always occur and that this dysfunction is not always the same. If the resection of the distal stomach causes hormonal changes, it would be expected to occur more frequently than observed. In contrast, after a distal gastrectomy, usually injury to the PEM or changes in the His angle do not occur. Therefore, these theories do not provide a reasonable explanation for their findings.

The study by Iida et al.^11^ is particularly intriguing and challenges the above theories. These authors measured LES resting pressure in 42 patients, before and after a distal gastrectomy, finding that in 21 (50%) patients, the LESP remained unchanged, while in 17 (40%) patients, the pressure decreased, and in 4 (10%) patients, there was a consistent increase in LESP. The authors describe these findings without giving a satisfactory explanation for their causes.

The hypothesis proposed and tested in this experimental study is based on the anatomical structure of the LES described by Liebermann-Meffert et al.^17^ and verified by studies of our group^12^ ^,^ ^13^. The LES is formed by two bands of muscles arranged almost perpendicularly which act in a complementary manner to close the cardia: the semicircular muscular fibers or “clasp” at the lesser curve and the oblique muscular fibers or “Sling fibers” on the side of the great curvature. The Sling fibers extend from the distal esophagus and proximal gastric fundus and run parallel to the lesser curve into the gastric antrum (Figure 1).

For this reason, the transverse transection of the upper third of the stomach or even in its middle third, by necessity, cuts the oblique “Sling” muscular fibers and, therefore, one of the sphincter components is severely damaged^9^ ^,^ ^12^ ^,^ ^13^ ^,^ ^17^ ^,^ ^18^ ^,^ ^21^ ^,^ ^22^. When gastrointestinal continuity is restored, via a gastroduodenal (Billroth I) or gastrojejunal anastomosis (Billroth II or Roux-in-Y), or the gastric remnant is closed (in this experimental study) by the suture, the oblique “Sling” fibers are reanchored. But this repair does not always restore either the symmetry or the tension of these fibers that existed before the transection and remnant closure, which can modify the LES resting pressure^22^.

Based on our results and the referred anatomical concepts, we maintain that it is possible to explain different effects on the LES sphincter, observed after distal gastrectomy^10^ ^,^ ^11^ ^,^ ^19^. The oblique fibers in patients can be reanchored with three possible outcomes: (1) the same symmetry and tension as before the gastric transection, thereby restoring the normal LESP and function; (2) with less tension creating a hypotensive, eventually incompetent sphincter; or (3) the fibers may be sutured with greater tension and tightness, thus resulting in a hypertensive sphincter.

Some limitations of this investigation are the low number of dogs (only seven dogs), acute experimental conditions, specimens under general anesthetic, and lack of a control group.

## CONCLUSION

Nevertheless, the findings of our experimental study in dogs appear to reproduce the clinical observations in men and give a reasonable explanation for different changes in LES resting pressure observed in patients after distal gastrectomy. We believe that these findings provide an anatomically and physiologically consistent answer to an old mystery of gastric surgery.
